# Environmental and behavioral changes may influence the exposure of an Arctic apex predator to pathogens and contaminants

**DOI:** 10.1038/s41598-017-13496-9

**Published:** 2017-10-16

**Authors:** Todd C. Atwood, Colleen Duncan, Kelly A. Patyk, Pauline Nol, Jack Rhyan, Matthew McCollum, Melissa A. McKinney, Andrew M. Ramey, Camila K. Cerqueira-Cézar, Oliver C. H. Kwok, Jitender P. Dubey, Steven Hennager

**Affiliations:** 1 US Geological Survey, Alaska Science Center, 4210 University Drive, Anchorage, AK 99508 USA; 2 Colorado State University, Department of Microbiology, Immunology and Pathology, 300 West Drake Ave., Fort Collins, CO 80523 USA; 3 United States Department of Agriculture/APHIS/VS/STAS/Center for Epidemiology and Animal Health, 2150 Centre Ave., Bldg. B, Fort Collins, CO 80521 USA; 4 United States Department of Agriculture/APHIS/VS/National Wildlife Research Center, 4101 Laporte Ave., Fort Collins, CO 80521 USA; 5 University of Connecticut, Wildlife and Fisheries Conservation Center, Department of Natural Resources and the Environment and the Center for Environmental Sciences and Engineering, 1376 Storrs Road, Unit 4087, Storrs, CT 06269-4087 USA; 6 United States Department of Agriculture, Agricultural Research Service, Beltsville Agricultural Research Center, Animal Parasitic Diseases Laboratory, Building 1001, Beltsville, MD 20705-2350 USA; 7 United States Department of Agriculture/APHIS/VS/STAS/National Veterinary Services Laboratory, Diagnostic Bacteriology Laboratory- Serology Section, 1920 Dayton Ave., Ames, IA 50010 USA

## Abstract

Recent decline of sea ice habitat has coincided with increased use of land by polar bears (*Ursus maritimus*) from the southern Beaufort Sea (SB), which may alter the risks of exposure to pathogens and contaminants. We assayed blood samples from SB polar bears to assess prior exposure to the pathogens *Brucella* spp., *Toxoplasma gondii, Coxiella burnetii*, *Francisella tularensis*, and *Neospora caninum*, estimate concentrations of persistent organic pollutants (POPs), and evaluate risk factors associated with exposure to pathogens and POPs. We found that seroprevalence of *Brucella* spp. and *T. gondii* antibodies likely increased through time, and provide the first evidence of exposure of polar bears to *C. burnetii*, *N. caninum*, and *F. tularensis*. Additionally, the odds of exposure to *T. gondii* were greater for bears that used land than for bears that remained on the sea ice during summer and fall, while mean concentrations of the POP chlordane (ΣCHL) were lower for land-based bears. Changes in polar bear behavior brought about by climate-induced modifications to the Arctic marine ecosystem may increase exposure risk to certain pathogens and alter contaminant exposure pathways.

## Introduction

Environmental conditions such as climate and landscape structure can influence the occurrence and spread of pathogens. Climatic factors such as precipitation, humidity, and air and water temperature have direct and indirect influences on survivorship, reproduction, and transmissibility of infectious agents such as viruses, bacteria, fungi, and parasites^1–3^. Landscape characteristics, including the juxtaposition of habitat types, availability of movement corridors, and distribution of critical resources, shape the behavior, distribution, and densities of host and vector species^4,5^. Thus, climate and landscape structure interact to govern the transmission dynamics of infectious agents, and may influence the health of susceptible host species. Environmental changes that alter the bio-climatic envelope of host and/or vector communities can modify infection dynamics resulting in new foci of transmission or novel sources of zoonotic infectious agents^6^. This may be particularly evident at areas experiencing rapid environmental changes such as those that have occurred recently in the Arctic. From 1989–2008, near-surface temperatures in the Arctic increased at a rate of 1.6 °C per decade in autumn, while sea ice extent declined at a rate of −7.9% per decade^7^. Concurrent with those environmental changes has been the emergence, re-emergence, and spread of various pathogens associated with Arctic wildlife^8–10^.

Polar bears (*Ursus maritimus*) are long-lived, range widely, and feed on a variety of marine mammal prey including ice seals (primarily ringed (*Pusa hispida*) and bearded (*Erignathus barbatus*) seals) and cetaceans (e.g., beluga whales (*Delphinapterus leucas*))^11,12^. Decline of sea ice habitat has altered the behavior of polar bears from some subpopulations, resulting in bears spending more time on land in summer and fall^13–15^. For example, polar bears from the southern Beaufort Sea (SB) subpopulation historically spent most of the year on sea ice^16^. Yet, recent studies and observations indicate that both the proportion of the subpopulation coming ashore and the duration of stay have increased since the accelerated decline in sea ice extent which began in the early 2000s^15,17–19^. Polar bears that use land in summer and fall may increase their cumulative risk of exposure to infectious agents and contaminants to also include those primarily associated with terrestrial habitats. Given that polar bears are likely to become more reliant on terrestrial habitats, an assessment as to whether such change is mediating an increased risk of exposure to infectious agents and contaminants would be informative.

Polar bears that come ashore do not have routine access to ice seals, but may have access to human-provisioned resource subsidies. Among bears in the SB subpopulation, this is mostly in the form of bowhead whale (*Balaena mysticetus*) carcasses remaining after harvest by Alaskan Natives^20,21^. Chemical tracer-based feeding estimates suggest that bowhead whale remains comprise 50–70% of land-based SB bear diets^12^. Polar bear presence and feeding at bowhead whale carrion sites overlaps in time and space with species associated with terrestrial and near-shore habitats such as Arctic foxes (*Vulpes lagopus*), grizzly bears (*Ursus arctos*), glaucous gulls (*Larus hyperboreus*), and ravens (*Corvus corax*). The common use of terrestrial habitats, and aggregations of wildlife at resource-rich sites, may increase intra- and inter-specific contact rates and result in an increased likelihood of transmission of infectious agents. Additionally, the dietary shift by terrestrial-based polar bears (i.e., those that come ashore when ice over the continental shelf is absent) from ice seals to bowhead whale remains, may influence exposure to contaminants and immune status^22,23^. Bowhead whales filter feed on zooplankton, while ice seals feed on fish and invertebrates^24^. As a result, bowhead whales occupy a lower trophic position than seals and may represent a less contaminated food source with respect to certain persistent organic pollutants (POPs)^25^.

We investigated risk factors influencing the seroprevalence of *Brucella* spp., *Coxiella burnetii*, *Toxoplasma gondii, Francisella tularensis*, and *Neospora caninum* among polar bears from Alaska’s southern Beaufort Sea. The focal zoonotic agents included in this study vary in geographic distribution, routes of transmission, and effects on wildlife. Exposures to *Brucella* spp. and *T. gondii* have previously been documented for a variety of Arctic marine mammals, including polar bears^26–28^. By contrast, exposures to *C. burnetii*, *F. tularensis*, and *N. caninum* have not been documented in marine mammals resident to the Arctic, though the latter two are pathogens found in high latitude terrestrial systems^29–32^. We were particularly interested in characterizing polar bear exposure to *C. burnetii* because it may be undergoing a northward range expansion in the marine environment from the Pacific Ocean into the Arctic Ocean^33,34^ and, like *Brucella* spp., can impair reproductive health and influence population natality rates^34^. Additionally, we characterized factors influencing circulating concentrations of major POPs (i.e., polychlorinated biphenyls [PCBs] and organochlorine pesticides [OCs]) in SB polar bears. These contaminants previously have been found at concentrations above general risk thresholds and risk quotients for reproductive, immune, and carcinogenic effects in this and other polar bear subpopulations^35,36^. By characterizing exposure risks to a suite of pathogens and POPs with different historical distributions and routes of transmission/exposure during a period of environmental change, we can gain insight into emerging health risks for polar bears and other Arctic wildlife species.

## Results

### Seroprevalence

In total, 161 samples were collected from 139 adult and subadult polar bears from 2007–2014 to test for exposure to the five pathogens. Of the 139 bears sampled, 22 were sampled 2–3 times over the period of the study. We tested 161 samples for antibodies to *C. burnetii, Brucella* spp. and *T. gondii*, 159 samples for antibodies to *N. caninum*, and 124 samples for antibodies to *F. tularensis*. Samples collected in 2014 were not tested for *F. tularensis* due to limited availability of serum. The number of serum samples used to calculate seroprevalence varied between 108 and 138 depending on the pathogen (Table 1). Antibodies to *C. burnetii* and *T. gondii* were detected most commonly, followed by *Brucella* spp., *F. tularensis*, and *N. caninum* (Table 1). For *C. burnetii* and *T. gondii*, individual seropositive titer levels varied substantially (Table 2). Only low levels of *F. tularensis* and *N. caninum* antibodies were detected, thus the biological significance of these titers is unknown.

**Table 1:** Seroprevalence, sample sizes, and 95% confidence intervals for selected pathogens in polar bears captured on the coast and sea ice of the southern Beaufort Sea, Alaska, USA, 2007–2014.

	*C. burnetii*	*T. gondii*	*Brucella* spp.	*F. tularensis*	*N. caninum*
seropositive (n)	38	33	18	5	5
seronegative (n)	100	105	120	103	133
mean seroprevalence	27.6%	23.9%	13.0%	4.8%	3.7%
95% CI	20.4–35.7	17.1–31.2	8.1–19.7	1.9–10.8	1.2–7.3

**Table 2:** Test method, seropositive titer threshold values, and the number of serum samples from subadult and adult polar bears assigned to each threshold bin based on test results.

Pathogen	Test method	Positive titer thresholds	n
*C. burnetii*	indirect fluorescence assay	1:128	24
		1:256	7
		1:512	3
		≥1:1024	4
*T. gondii*	modified agglutination test	1:50	2
		1:100	4
		≥1:200	27
*F. tularensis*	slide agglutination test	1:20	3
		1:40	2
*N. caninum*	Neospora agglutination test	1:25	3
		1:50	2

Polar bears were captured on the coast and sea ice of the southern Beaufort Sea, Alaska, USA, 2007–2014.

Evidence for prior exposure to more than one pathogen was rare, with co-occurring antibodies (or evidence for cross reactivity of assays) detected for *C. burnetii* and *T. gondii* (2.9%), *C. burnetii* and *Brucella* spp. (2.2%), *T. gondii* and *N. caninum* (1.5%), *Brucella* spp. and *T. gondii* (1.4%), *Brucella* spp. and *F. tularensis* (0.9%), and *F. tularensis* and *N. caninum* (0.9%).

### Risk factors of pathogen exposure

Age class was most strongly associated with exposure to *C. burnetii* (Table 3). The odds ratio for an age effect indicated that adult polar bears were 3.2 times more likely to be seropositive than subadults. Sex and year of capture were included in two additional models that formed the top set though offered less support (Table 3); however, 85% confidence intervals for sex class and year of capture overlapped zero, indicating these variables may be uninformative^37^ (Table 4). The four models in the candidate model set accounted for 70% of all model weight, with the top model having a weight of evidence 1.5 to 2.1 times greater than other candidate models (Table 4). All other models characterizing factors mediating exposure to *C. burnetii* had ΔAIC_c_ >2 (Table 3) and no variables from any model had a *p*-value < 0.05 (Table 4).

**Table 3:** Models evaluated and selection results for generalized linear mixed models used to evaluate factors influencing exposure to *C. burnetii*, *T. gondii*, and *Brucella* spp. of adult (≥5 yrs old) and subadult (2–4 yrs old) polar bears captured on the coast and sea ice of the southern Beaufort Sea, Alaska, USA, 2007–2014.

Pathogen	Models	K	AIC_c_	Δ AIC_c_	Akaike Wt. (*w*_i_)
*C. burnetii*	intercept + age	3	174.74	0.00	0.26
	intercept + age + sex	5	175.61	0.87	0.17
	intercept + year of capture	9	175.97	1.23	0.14
	intercept + sex	3	176.24	1.50	0.12
	intercept + age + habitat use	5	176.82	2.08	0.09
	intercept + habitat use	3	177.57	2.83	0.06
	intercept + sex + habitat use	5	177.92	3.18	0.05
	intercept + year of capture + habitat use	11	178.04	3.30	0.05
	intercept + year of capture + habitat use + sex + age	15	178.42	3.68	0.04
*T. gondii*	intercept + habitat use	3	153.80	0.00	0.39
	intercept + sex + habitat use	5	154.92	1.12	0.22
	intercept + age + habitat use	5	155.20	1.40	0.19
	intercept + year of capture + habitat use	11	155.83	2.03	0.14
	intercept + year of capture + habitat use + sex + age	15	158.06	4.26	0.05
	intercept + age + sex	5	171.42	17.62	<0.01
	intercept + sex	3	172.80	19.00	<0.01
	intercept + age	3	174.74	20.94	<0.01
	intercept + year of capture	9	175.97	22.17	<0.01
*Brucella* spp.	intercept + sex + habitat use	5	118.75	0.00	0.17
	intercept + year of capture + habitat use	11	119.18	0.43	0.14
	intercept + sex	3	119.22	0.47	0.14
	intercept + habitat use	3	119.53	0.78	0.12
	intercept + year of capture	9	119.55	0.80	0.11
	intercept + age	3	119.87	1.12	0.09
	intercept + year of capture + habitat use + sex + age	15	120.18	1.43	0.08
	intercept + age + sex	5	120.37	1.62	0.07
	intercept + age + habitat use	5	120.93	2.18	0.06

Akaike’s Information Criterion corrected for small sample size (AIC_c_) and ΔAIC_c_ were used to identify top model sets. Normalized Akaike weights (*w*_*i*_) were used to assess individual model information content (models with ΔAIC_c_ values ≤2.0 were considered to provide similar levels of empirical support).

**Table 4:** Coefficient estimates for the top-ranked models (i.e., models with Δ AIC_c_ ≤ 2.0) of factors influencing exposure to *C. burnetii*, *T. gondii*, and *Brucella* spp. of adult (≥5 yrs old) and subadult (2–4 yrs old) polar bears captured on the coast and sea ice of the southern Beaufort Sea, Alaska, USA, 2007–2014.

Pathogen	Model Rank	Explanatory Variables	Estimate (β) (85% CI)	S.E.	P-value	Odds Ratio (95% CI)
*C. burnetii*	1	age	1.16 (0.03–2.28)	0.78	0.148	3.18 (0.65–15.63)
	2	age	1.13 (0.01–2.25)	0.78	0.16	3.08 (0.62–15.21)
		sex	0.41 (−0.15–0.97)	0.39	0.308	1.51 (0.67–3.38)
	3	year of capture	−0.09 (−0.19–0.01)	0.07	0.205	0.91 (0.78–1.06)
	4	sex	0.44 (−0.12–1.00)	0.39	0.267	1.55 (0.70–3.45)
*T. gondii*	1	habitat use	1.95 (1.27–2.63)	0.47	<0.001	7.01 (2.66–18.49)
	2	habitat use	1.87 (1.18–2.56)	0.48	<0.001	6.51 (2.42–17.56)
		sex	−0.35 (−1.06–0.36)	0.49	0.482	0.71 (0.26–1.92)
	3	habitat use	1.88 (1.19–2.57)	0.48	<0.001	6.57 (2.45–17.66)
		age	0.65 (−0.62–1.92)	0.88	0.47	1.91 (0.31–11.62)
*Brucella* spp.	1	sex	−0.87 (−1.66– −0.78)	0.55	0.123	0.42 (0.14–1.29)
		habitat use	−0.86 (−1.71– −0.01)	0.59	0.154	0.42 (0.13–0.14)
	2	habitat use	−0.86 (−1.71– −0.01)	0.59	0.156	0.42 (0.13–1.42)
		year of capture	−0.15 (−0.28– −0.02)	0.09	0.148	0.38 (0.70–1.06)
	3	sex	−0.65 (−1.39–0.09)	0.52	0.221	0.52 (0.18–1.52)
	4	habitat use	−0.63 (−1.44–0.02)	0.56	0.277	0.54 (0.17–1.70)
	5	year of capture	−0.11 (−0.24–0.02)	0.09	0.27	0.89 (0.73–1.09)
	6	age	−0.68 (−1.61–0.24)	0.64	0.303	0.51 (0.13–1.91)
	7	sex	−0.75 (−1.56–0.06)	0.56	0.196	0.47 (0.15–2.39)
		habitat use	−0.97 (−1.88– −0.06)	0.63	0.131	0.38 (0.11–1.36)
		year of capture	−0.15 (−0.29– −0.01)	0.1	0.151	0.86 (0.69–1.06)
		age	−0.59 (−1.61–0.43)	0.71	0.415	0.55 (0.13–2.39)
	8	sex	−0.62 (−1.38–0.14)	0.53	0.249	0.54 (0.18–1.59)
		age	−0.62 (−1.56–0.32)	0.65	0.354	0.54 (0.14–2.06)

For *T. gondii*, three models were included in the top model set (Table 3), with all including an effect of habitat use (i.e., bears spending summer on shore or on the sea ice). Top models also included an additive effect of age class and sex (Table 3). The odds ratio for habitat use indicated that the odds of polar bears using land during summer being seropositive to *T. gondii* were 7 times greater than the odds of bears remaining on the sea ice during summer being seropositive (Table 4). The 85% confidence intervals for sex class and age class overlapped zero, indicating the variables may be uninformative^37^ (Table 4). The three models in the candidate model set accounted for 81% of all model weight, with the top model having a weight of evidence 1.7 to 2.0 times greater than the other candidate models (Table 3). The remaining models characterizing exposure to *T. gondii* had ΔAIC_c_ >2 (Table 3).

Although >10% of individuals were seropositive for *Brucella* spp., we were unable to identify a top model for characterizing risk factors of exposure (Table 3). The two top-ranked models included an effect of habitat use, with the odds ratio indicating that the odds of polar bears using land during summer being seropositive for *Brucella* spp. were 2.5 times lower than the odds of bears remaining on the sea ice during summer being seropositive. The 85% confidence intervals for the sex class and capture year variables in the two top-ranked models did not overlap zero, though they did in lower-ranked models. However, weight of evidence indicated no clear top model or set, with 68% of all model weight distributed among 5 of the 9 models (Table 3). No model variables had a *p*-value < 0.05 (Table 4). We did not characterize risk factors of exposure to pathogens to *F. tularensis* and *N. caninum* because both had <10% seropositive individuals.

### Contaminant concentrations

Samples collected from 52 seropositive individuals in 2013 and 2014 were used to quantify exposure to POPs. Mean concentrations of ΣPCB did not vary by sex (*F*_1,50_ = 1.32, *p* = 0.12) or habitat use (F_1,50_ = 0.19, P = 0.56) (Table 4). Results of OC analyses differed by compounds. Mean concentrations of ΣClBz were higher for females (sex: F_1,51_ = 0.70, P = 0.05) but did not differ relative to habitat use (F_1,51_ = 0.01, P = 0.78). Mean concentrations of ΣHCH were higher for males and also did not differ relative to habitat use (sex: F_1,51_ = 3.68, P = 0.006; habitat use: F_1,51_ = 0.04, P = 0.77). Mean concentrations of ΣCHL varied by sex (F_1,51_ = 3.25, P < 0.001) and habitat use (F_1,51_ = 0.59, P = 0.05) (sex × habitat use: F_1,51_ = 0.79, P = 0.03), with concentrations higher for females and individuals that remained on the sea ice during summer (Table 5). Mean concentrations of ΣDDT did not vary by sex (F_1,51_ = 0.04, P = 0.40) or habitat use (F_1,51_ = 0.12, P = 0.13). No other interaction terms were significant. The small sample of subadults precluded assessment of age class as an effect in ANOVAs.

**Table 5:** Concentrations of ΣPCB, ΣClBz, ΣHCH, ΣCHL, and ΣDDT (ng g^−1^ wet weight) by age class, sex, and habitat use (on land or on sea ice during summer) of southern Beaufort Sea polar bears, captured on the sea ice 2013–2014, Alaska, USA.

	n	ΣPCB	95% CI	ΣClBz	95% CI	ΣHCH	95% CI	ΣCHL	95% CI	ΣDDT	95% CI
		Mean (SE)		Mean (SE)		Mean (SE)		Mean (SE)		Mean (SE)	
**sex**											
male	22	12.74 (1.53)	9.73–15.75	1.60 (0.36)	0.90−2.30	2.58	1.73–3.43	9.08	6.48–11.68	0.15	−0.01–0.31
						−0.43		−1.33		−0.08	
female	30	12.27 (2.50)	7.36–17.18	2.03 (0.22)	1.59−2.47	1.39	0.61–2.17	14.56	12.73–16.39	0.12	−0.01–0.25
						−0.39		−0.93		−0.06	
**habitat use**											
onshore	11	9.98 (1.52)		1.59 (0.20)		1.73	0.65–2.81	10.04	7.04–13.04	0.23	0.002–0.46
			7.00–12.96		1.19−1.99	−0.55		−1.53		−0.11	
sea ice	41	13.39 (1.83)		1.94 (0.25)		1.95	1.25–2.65	12.98	11.02–14.94	0.10	−0.01–0.21
			9.80–16.98		1.45−2.43	−0.36		−1.00		−0.05	

## Discussion

We provide evidence: (i) of a possible increase in seroprevalence to *Brucella* spp. and *T. gondii* in SB polar bears, (ii) of the potential exposure of SB polar bears to *C. burnetii*, *N. caninum*, and *F. tularensis*, and (iii) that previously-documented climate-induced change in polar bear behaviors^12,15^ may have influenced exposures to certain POPs and disease agents. Collectively, these findings help elucidate the way in which ecologically-driven behavioral change may alter exposure risks posed by environmental stressors.

Our analyses suggest that the seroprevalence of both *T. gondii* and *Brucella* spp. increased over the last decade. Prior studies of the SB subpopulation reported overall prevalence of 13.2% for *T. gondii* from 2005–2006^38^ and 10.2% for *Brucella* spp. from 2003–2006^26^. It is important to note that the previous *Brucella* study^26^ used the same screening test as this study, while the earlier *T. gondii* work^38^ used a latex agglutination assay and a positive titer cut-off of >1:16 (rather than the MAT with positive titer cut-off of >1:25). Further, the 95% CI for *Brucella* spp. antibodies from the present study overlaps the point estimate from the prior study^26^. Given those caveats, our study suggests that over the subsequent eight years, overall seroprevalence of *T. gondii* may have increased by 81%, while overall prevalence of *Brucella* spp. may have increased by 27%. The reasons for these potential increases are unclear. There is some evidence to suggest that exposure to *T. gondii*, which has previously been reported as infecting various Arctic terrestrial mammals^39^, has become more prevalent in the marine environment. For example, there is evidence of exposure to *T. gondii* for ringed and bearded seals sampled in the Svalbard archipelago^27^, and in ringed seals throughout the Canadian Arctic^40^. Unlike *T. gondii*, *Brucella* spp. has long been recognized as infecting both terrestrial and marine hosts inhabiting the Arctic (e.g., caribou, muskox, pinnipeds, and cetaceans^28,41–43^). Our finding of a likely increase in exposure to these pathogens for SB polar bears, concurrent with observations in a variety of Arctic species, suggests that there may be multiple routes of exposure to these pathogens.

Changes that have occurred to the Arctic marine ecosystem over the last two decades have been posited as a potential factor altering host-pathogen interactions^44^. Modified environmental conditions, such as the protracted open-water period (i.e., period of time when sea ice concentration over the continental shelf is <15%) during summer and fall, have led to increases in ocean temperature and primary production^38,45,46^. These changes may be facilitating a northward range expansion for some subarctic pathogens and alterations in transmission dynamics. For example, *C. burnetii*, a widely distributed pathogen, has recently been detected in several species of marine mammals^33,34^, with the previous northernmost case reported for northern fur seals (*Callorhinus ursinus*) from the Pribilof Islands in the Bering Sea^34^. Our finding of evidence of polar bear exposure to *C. burnetii*, a linear distance of 1,738 km from the Pribilof Islands, marks the first detection of exposure for an Arctic marine mammal. However it remains unclear if this is a function of northward expansion of *C. burnetii*, attributable to long-distance migratory behavior of some polar bears^47,48^, a lack of surveillance in marine mammals of the Arctic, or detection of an unknown pathogen that cross-reacts with the test. With regard to *F. tularensis* and *N. caninum*, these pathogens may be considered terrestrial-based pathogens in that *F. tularensis* has long-been associated with lagomorphs and *N. caninum* is primarily a pathogen of cattle, some species of deer, and dogs^30^. Although antibodies to *N*. *caninum* were reported in some herbivores and canids from Alaska^32,49^, and *N. caninum* DNA has been detected in tissues of 11 (24.4%) of 45 European brown bears (*Ursus arctos*) from Slovakia^50^, this is the first report of exposure to *N. caninum* in polar bears in Alaska^51^. Similarly, while *F. tularensis* antibodies have previously been reported in 14–28% of grizzly bears inhabiting the Alaskan Arctic^52^, no comparable assessments have been made for polar bears in this region. The apparent detection of seropositive polar bears also represents the first evidence for potential *F. tularensis* and *N. caninum* exposure, to our knowledge, for Arctic marine mammals.

### Risk factors associated with exposure to pathogens and contaminants

Since 2000, the proportion of the SB polar bear subpopulation coming ashore during summer and fall has increased from 6% to over 20%, and the length of stay on land has increased by over a month^15^. While on land, most polar bears visit sites where subsistence-harvested bowhead whale remains are aggregated, and may spend several weeks feeding on the carrion^12,20^, which may increase the risk of density-dependent inter- and intra-specific pathogen transmission^53^. In modeling factors influencing exposure risk, we found evidence to support the notion that land use, including visiting sites of whale remains, may increase the risk of exposure to *T. gondii* and decrease risk of exposure to *Brucella* spp. Oocysts of *T. gondii* have high environmental resistance and have been shown to persist in soil in temperate environments^54^. However, it is unknown whether they may be able to persist in the soil where whale remains are aggregated and contribute to a chronic risk of exposure. With regards to *Brucella* spp., some have posited that the exposure pathway may be terrestrial, while others questioned that conclusion^26,55^. Our results suggest there may be a lower risk of exposure to *Brucella* spp. for polar bears that use terrestrial habitats. However, we note that while our work suggests that bears remaining on the sea ice year-round had a tendency towards a higher likelihood of exposure to *Brucella* spp., we did not differentiate exposures to the various strains of *Brucella* spp. which would be useful for identifying transmission pathways.

We found no influence of age and sex classes on risk of exposure to *T. gondii* and *Brucella* spp., which is similar to previous studies in the SB but differs from studies elsewhere. For example, there was no difference by sex and age class in the prevalence of *T. gondii* for SB polar bears that were sampled in 2005 and 2006^38^, and for individuals exposed to *Brucella* spp. from 1982–1999^56^. Conversely, the prevalence of *T. gondii* in polar bears from the Svalbard archipelago was significantly higher in adult males compared to adult females^27^. Additionally, the prevalence of *T. gondii* was higher for bearded seals compared to ringed seals in Svalbard suggesting that the difference in polar bear prevalence may result from adult male bears being more likely to prey on the larger-bodied bearded seals than adult female bears^27^. We did find that adult polar bears were more likely than subadults to be exposed to *C. burnetii*, though the reason for the difference is not clear. Unlike the Svalbard study^27^, we lacked information on the prevalence of pathogens for prey species in the SB. Antibodies to *C. burnetii* may be long-lived and therefore higher prevalence in adults may reflect cumulative exposure to this bacterium through time. However, while both models from the top model set of exposure to *C. burnetii* contained the age class explanatory variable, the coefficient estimates were not statistically significant and biological significance should be interpreted cautiously.

Co-infections are common in wildlife populations and can influence infection risk^57^ and host fitness^58^. Host immune suppression caused by one pathogen may increase risk of infection from another pathogen^59^, and pathogen-pathogen interactions may have additive effects on host health^60^. We found that possible co-exposures among the five pathogens were rare (i.e., <3%). Several lines of evidence suggest polar bears from the SB may be exposed to multiple stressors capable of undermining population health. For example, gene transcription profiles of bears sampled in the SB and adjacent Chukchi Sea during the later years of our study indicated immune function impairment for SB bears^61^ and, in complimentary work involving a subset of the same individuals, a signal of enhanced viral defense was detected^62^. Additionally, the probability of fasting by SB polar bears in spring has increased over time^63^, suggestive of a greater frequency of periods of nutritional stress. Future work should focus on characterizing the co-exposure of polar bears to numerous stressors including viruses and the potential for synergistic effects of co-infection, impaired immune function, and nutritional restriction on population dynamics. Because increased contaminant burdens in marine mammals also can alter the function of physiological pathways associated with metabolism and immunological response^64,65^, they too should be included in assessments of multiple stressors on polar bear population dynamics.

## Conclusions

Climate change-driven declines in sea ice habitat appear to be linked to increased use of land^15^, which may influence exposure to some pathogens and contaminants by SB polar bears. For example, we found that exposure to *Brucella* spp. and mean concentrations of ΣCHL were lower for bears that used terrestrial habitat. As noted above, the reason for reduced exposure to *Brucella* spp. is unknown, but reduced concentrations of CHLs in land-based bears likely are mediated by a shift from foraging predominantly on ringed seals to scavenging bowhead whale carcasses^12,66^. Polar bears from the Beaufort Sea region have historically preyed primarily on ringed and bearded seals and, to a lesser degree, beluga whales, which all have diets that include both fish and invertebrates^24,67^. As a result, seals and beluga whales occupy a relatively high trophic position and are vulnerable to the bioaccumulation and biomagnification of harmful contaminants like POPs. By contrast, bowhead whales filter feed zooplankton which occupy a lower trophic position than fish and invertebrates. Thus bowheads should be a less contaminated food source with respect to exposure to strongly biomagnifying POPs like CHLs for polar bears. Lower concentrations of other OC pesticides may be less expected in onshore bears as some of the OCs do not biomagnify to the same extent as CHLs, and have been shown to be at a similar or even higher concentration (e.g., ClBzs, HCHs, DDTs) in bowhead whales relative to ringed and bearded seals in this region^68^.

The sources and consequences of exposure of polar bears to the pathogens and contaminants we surveyed remain largely unknown. Both *C. burnetii* and *Brucella* spp. are associated with reproductive pathology in a wide range of species^69^. The population-level effects of toxoplasmosis in wildlife are unknown though it has been linked to compromised immune function^70^, as has recently been observed in SB polar bears^61^. While there is no evidence of population-level effects of *F. tularensis* and *N. caninum* in large carnivores^71,72^, tularemia epizootics are capable of regulating small mammal populations^73,74^ and neosporosis can cause high abortion rates in cattle and neurological disease in dogs^75^. Thus, all of the infectious agents we surveyed have the potential to threaten polar bear population health, which is a concern given their status as “vulnerable” under the International Union for the Conservation of Nature (IUCN) Red List Assessment. Causal investigations are needed to understand the potential impacts that these agents may have on polar bear population dynamics.

## Methods

### Study area and data collection

**Figure 1:**
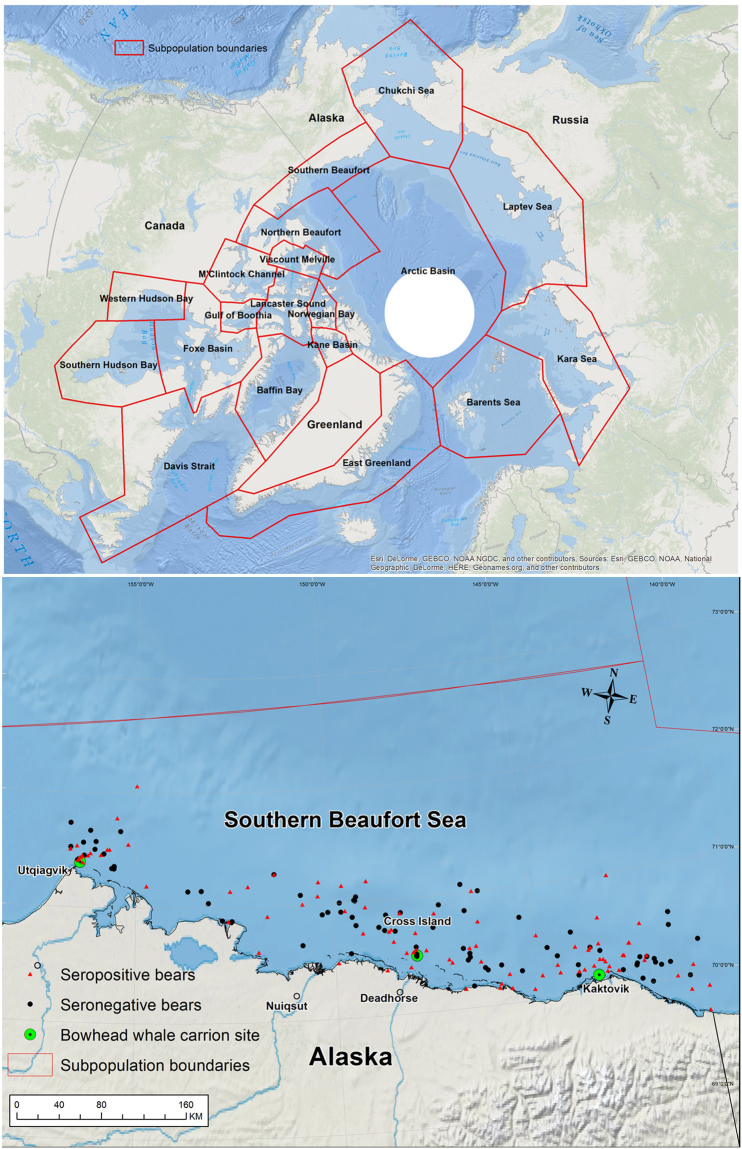
The upper panel contains a map of the 19 polar bear subpopulation units recognized by the International Union for the Conservation of Nature/Polar Bear Specialist Group. The lower panel contains capture locations of polar bears used to assess patterns of exposure to infectious agents and contaminants, southern Beaufort Sea, Alaska, USA, 2007–2014. Positive bears are individuals that were seropositive for antibodies to at least one of the five pathogens surveyed. The map was created using ArcMap 10.4 (http://desktop.arcgis.com/en/arcmap/).

The study area was the Alaska portion of the SB, ranging from Demarcation Point (69°N, 141° W) at the US−Canada border in the east to Point Barrow (71°N, 156° W) in the west (Fig. 1). The Beaufort Sea is characterized by a narrow continental shelf, beyond which is some of the deepest water in the Arctic Ocean^76^. The character and spatio-temporal extent of sea ice in the Beaufort Sea has changed substantially since 1979 (i.e., advent of satellite observation of sea ice). Historically, sea ice was persistently available over or adjacent to the continental shelf. However, since the early 2000s, the duration of the open-water period has increased at a rate of ~9 days per decade, which is among the largest rates of increase for the seas of the Arctic Ocean^77,78^.

The SB coastal region is characterized by an industrial footprint associated with oil and gas exploration and extraction, generally concentrated along the central coast^79^. There are three communities within the study area that harvest bowhead whales in the fall: Utqiagvik (formerly known as Barrow), Nuiqsut, and Kaktovik (Fig. 1). Remains from the harvest have been sporadically aggregated at Point Barrow (up to 2012) and consistently aggregated at Cross Island and Barter Island (Fig. 1). These locations have served as focal attractors for polar bears during fall^15,17,21^.

Polar bears were encountered from a helicopter opportunistically on the sea ice from mid-March to mid-May, 2007–2014, and on land in August and October of 2008 and 2009. Bears were immobilized with the drug tiletamine hydrochloride plus zolazepam hydrochloride (Telazol^®^, Fort Dodge and Warner-Lambert Co.) using a projectile syringe fired from a dart gun. Captured bears were uniquely identified using ear tags and a corresponding lip tattoo. A subset of adult females was fitted with global positioning system (GPS) satellite radio collars. Age at first capture of subadults and adults was determined by analysis of cementum annuli from a vestigial premolar, while dependent young were aged visually^80,81^. Ear punch samples for all captured individuals were genotyped at up to 20 microsatellite loci by Wildlife Genetics International (Nelson, British Columbia, Canada).

Blood samples from subadult and adult bears were drawn into additive-free and EDTA-treated evacuated tubes by venipuncture of either the femoral or jugular veins. All samples were centrifuged at 3,500 rpm for 5 minutes on the day of collection to derive sera and plasma^38^, aliquoted into 2 mL cryovials, stored at −20 °C, and then stored at −80 °C upon return from the field. Each serum sample was tested for antibodies to *Brucella* spp., *C. burnetii*, *T. gondii, F. tularensis*, and *N. caninum*. Plasma samples from a subset of individuals that had antibodies to one or more of these pathogens were used to quantify exposure to PCBs and OC pesticides.

The buffered *Brucella* antigen card test and standard plate test (SPT) were performed at the National Veterinary Services Laboratory in Ames, IA. A sample was considered positive if either the card test or SPT was positive. *C. burnetii* indirect fluorescence assay (IFA) was conducted^33^ at Colorado State University in Fort Collins, CO, with a cut-off titer of 1:128 considered positive for prior exposure.

Serological testing for *T. gondii* and *N. caninum* was performed at the Animal Parasitic Diseases Laboratory (APDL), Beltsville Agricultural Research Center, Beltsville, MD. The modified agglutination test (MAT) was used for the detection of *T. gondii* with a cut-off titer of 1:25^82,83^. The MAT is considered highly specific and sensitive, can be used for all host species, and has been extensively validated using isolation of *T. gondii* for several species, including black bears^84^. For *N. caninum* testing, a similar agglutination test was used (*Neospora* agglutination test, NAT)^85^.

To test polar bears for prior exposure to *F. tularensis*, a commercial slide agglutination test was performed at the U.S. Geological Survey Alaska Science Center, Anchorage, AK, according to the manufacturer’s protocol (Becton, Dickinson and Company, Sparks, MD), using serial dilutions of 1:20–1:320. *F. tularensis* antisera were tested in parallel with all polar bear sera to serve as positive controls. Polar bear sera reacting to antigen at a 1:20 or higher dilution were considered as putatively positive for prior exposure to *F. tularensis*. Thirty-three percent of samples were run in duplicate as quality control.

Sample extraction and analysis to assess exposure to PCBs and OCs were performed at the Center for Environmental Science and Engineering (CESE) at the University of Connecticut, Storrs, CT, according to established protocols^86,87^, with modifications as described. Approximately 1.5 g of blood plasma was weighed and spiked with the following deuterated surrogate standards: 1,2,4,5-tetrachlorobenzene (1,2,4,5-tetraClBz), and CBs 9, 116 and 156. Samples were mixed and allowed to equilibrate for 30 min., 1 mL of 6 M hydrochloric acid (HCl) was added and mixed, then 3 mL of 2-propanol was added and mixed. A 6 mL aliquot of a 1:1 mixture of methyl t-butyl ether (MtBE):hexane was added, mixed, and followed by ultrasonication for 20 min., then centrifugation at 1000 rpm for 10 min. The organic phase was collected, and the extraction was repeated two further times. To the combined extracts, 6 ml of 1% potassium chloride was added and mixed, proceeded by centrifugation for 5 min @ 1000 rpm and subsequent collection of the organic phase. The extract was then concentrated and subject to further clean up by solid phase extraction (SPE)^86^. Finally, the eluate was exchanged into trimethylpentane, spiked with the internal standards, *o*-terphenyl and *m*-xylene, and concentrated to a final volume of 400 μl.

Concentrated extracts were monitored and quantified for a suite of PCBs and OCs as described elsewhere^87^. Extracts were run on a gas chromatograph coupled with a Quattro Micro tandem mass spectrometer (GC-MS/MS) system on a Rxi-5Sil GC column (30 m length column of 0.25 mm I.D., 0.25 µm film thickness (Restek Corporation, PA)). Waters MassLynx software v. 4.1 (Milford, MA) was used for data acquisition and processing. PCBs were monitored by multiple-reaction monitoring (MRM), while OCs were monitored by selected ion monitoring (SIM). Samples were analyzed for 40 individual or co-eluting PCB congeners: CBs 18, 31/28, 44, 47/48/49/52, 66, 70/76, 74, 85, 87, 95, 99, 101/90, 105, 110, 118, 128, 130, 138, 146, 149, 151, 156, 157, 153, 170/190, 179, 180, 183, 184, 187, 195, 206, and 209. The OC pesticides included the ClBzs: 1,2,4,5-tetraClBz, 1,2,3,4-tetraClBz, PeClBz and hexachlorobenzene (HCB); hexachlorocyclohexanes (HCH): α-HCH, β-HCH; chlordanes (CHL): *cis*-nonachlor, *trans*-nonachlor, *cis*-chlordane, *trans*-chlordane, oxychlordane, heptachlor and heptachlor epoxide; aldrin; endosulfan: α-endosulfan, β-endosulfan; DDTs: *p,p*-DDE, *p,p*-DDD, *p,p*-DDT; dieldrin, mirex: mirex, photomirex; and, methoxychlor. Contaminant concentrations were reported on a mg kg^−1^ basis. For ClBzs, but not other OCs or PCBs, reported concentrations were recovery-corrected.

Quality control steps included method blanks and spiked clean dog plasma extracted with each batch of samples. Method blanks were below the detection limit for PCBs and OCs. Accuracy and precision was shown by the *n* = 4 spiked dog plasma replicate ΣPCB and ΣOC concentrations of 92 ± 7% (±SD) and 84 ± 5%, respectively, of the spiked values (excluding methoxychlor, for which there appeared to be contamination or interference in the dog plasma, but not in the samples or blanks). In the samples, surrogate standard recoveries were 82 ± 12% for 1,2,4,5-tetraClBz and 83 ± 7% the PCBs. Instrument blanks, recovery standards and calibration standards were also included at the start of each run and after every 15 samples.

### Statistical analyses

We used multiple methods to differentiate between polar bears that spent time on shore (i.e., “onshore” bears) and on the sea ice (i.e., “offshore” bears) in the summer and fall prior to their spring capture. We used location data from radio-collared adult females to confirm land or sea ice use in summer and fall^15^. Additionally, individuals were classified as onshore bears if they were detected (by genetic identification) at hair-snags erected in the fall around the remains of bowhead whales at Point Barrow (2010–2011)^20^ or Kaktovik (2012–2014) or from biopsy-darting during fall coastal surveys from 2010–2013. Last, we classified bears as onshore based on the detection of bowhead whale stable isotope (SI) and fatty acid (FA) signatures in hair and fat samples collected during spring capture, 2007–2012^12,66^. Hair and fat biopsy samples were genotyped at 20 microsatellite loci and compared to genotypes from captured bears to identify individuals sampled at hair-snags and during aerial surveys. We used a threshold of >5% bowhead whale composition of the diet to classify an individual as being onshore. Bowhead whales of the Bering-Chukchi-Beaufort population typically winter in the Bering Sea, migrate along the coast northward to the Chukchi and Beaufort seas in spring, and migrate back to the Bering Sea in the fall^88^. As a result, bowhead whales, which are too large for polar bears to hunt, are only regularly available as a food source to polar bears scavenging on land in summer and fall. Thus detection of a SI or FA signature in spring is assumed to be indicative of bowhead whale consumption in the prior fall^12^. A bear was considered to be onshore if it was identified using any of the three previously-mentioned methods. There were no instances in which the classification techniques (i.e., radio-collar, hair-snag, and diet) yielded conflicting results.

For seroprevalence calculations, individuals that were captured multiple times during the study were only counted once and considered seropositive for a given infectious agent if any sample from an individual was positive. This approach was used to minimize overestimates of seroprevalence resulting from long-lived antibodies in individuals recaptured during the course of this study^89^. Observed seroprevalence and associated 95% confidence intervals (CI) were calculated using the package “epiR”^90^ in the R software, version 3.2.1 (R Development Core Team 2015).

For pathogens for which seroprevalence was ≥10% of individuals tested, we characterized factors influencing exposure using generalized linear mixed models (GLMM) with a binomial (logit) link. Prior exposure (positive or negative) of individuals to each pathogen was used as the response variable and year of sampling, sex, age (adult and subadult), and summer habitat use (onshore or sea ice) as explanatory variables. We included individual identity as a random effect to control for repeated observations of some individuals. We created *a priori* candidate models derived from combinations of explanatory variables and compared them using Akaike’s Information Criterion corrected for small sample size (AIC_c_) to aid in determining top models. We used AIC_c_ to rank and compare models based on ΔAIC_c_ and normalized Akaike weights *w*_*i*_ and considered models with ΔAIC_c_ values >2.0 to differ in information content^91^. When faced with model uncertainty, we reported all models, calculated 85% CI for parameter estimates, and considered parameters whose 85% CI overlapped zero to be uninformative^37^.

Exposure to PCBs and OCs were characterized from plasma samples collected in 2013 and 2014 from a subset of individuals used to calculate seroprevalence. Contaminants like PCBs and OCs can compromise the function of the immune response and increase host susceptibility to infectious pathogens^92,93^. We hypothesized that contaminant concentrations, and perhaps susceptibility to some pathogens, would be lower for individuals that spent time on land feeding on bowhead whale remains. To investigate this, we summed individual concentrations of PCBs and OCs and tested for differences in mean concentrations relative to sex class, habitat use, and their interaction using 2-way factorial ANOVAs. Statistical significance was accepted at α ≤ 0.05. All contaminant concentrations were log(x + 1) transformed to approximate normal distributions.

This research was approved under the Marine Mammal Protection Act and Endangered Species Act with U.S. Fish and Wildlife Service permit number MA690038. Capture protocols were approved by the U.S. Geological Survey Institutional Animal Care and Use Committee. All methods were performed in accordance with relevant guidelines and regulations.

### Data availability

Data have been publicly archived at https://alaska.usgs.gov/portal/.
